# Hospital for Sick Children

**Published:** 1871-05

**Authors:** 


*Hospital for Sick Children.* Notes of a Clinical Lecture on Chorea. By Dr. Dickinson.

[From the London Lancet, April 15.]

Dr. Dickinson commenced his lecture by giving a brief account of the affections closely allied to chorea, which have attracted attention at different times, and acquired separate names—namely, the dancing mania of St. John, which spread from Aix-la-Chapelle to Cologne, Metz, and Strasburg; the Tarantula dance of Southern Italy; the "Convulsionnaire" of France; and a similar affection which is observed among the natives of Abyssinia. He showed that these diseases were dependent on an intimate connexion between the intellectual faculty and the voluntary muscles, and that the infection spread mainly by imitation. He also suggested that the phenomena observed in the Jumpers of Cornwall and the Shakers of the New World, to follow the reception of powerful religious impressions, are likewise allied to chorea.

Directing attention to the *cause* of chorea, he said that it could, in a large number of instances, be traced to fright, and adduced the evidence of a number of cases which he had brought down from the wards for purposes of demonstration. Another, but less frequent, cause was irritation, arising from an unhealthy condition of some part of the body, such as a loaded intestine or an irritable condition of the uterus. A case in point was that of a girl sixteen years of age, who had died of an attack of chorea. The cervix uteri was found to be in a state of intense congestion, and the os was covered with a membrane resembling that of a diphtheria; there were also traces of recent endocardial deposit. The pathological connexion between chorea and certain conditions of the heart and lining membrane Dr. Dickinson was inclined to attribute to a peculiar condition of blood, which is favorable to the deposition of lymph, and tends also to induce congestion of the cerebro-spinal system. With regard to the age at which chorea most frequently occurs, the hospital registration was said to show that children between the ages of six and fourteen are most liable to it; but it was not purely a disease of childhood, though after puberty the frequency of its occurrence decreased with advancing years. One case which Dr. Dickinson mentioned was that of an old man, sixty-four years of age, who became violently affected on learning the death of his master.

Turning to the *symptoms* of the disease, Dr. Dickinson said that it had been well defined as an insanity of the voluntary muscles; the only involuntary muscle liable to be included in the morbid influence was the heart, which was histologically a near ally of the voluntary muscles. He thought that, nevertheless, the heart is not usually affected primarily like the other muscles, but influenced secondarily by the endocardial deposit. As to the parts chiefly affected, he said that, as would be expected, they were those which are principally governed by the centres in which the intellectual faculties preside, and that accordingly the muscles of the upper extremity were first and chiefly affected, those of the lower extremity being later affected, and to a less degree, and recovering earlier. Examples were then given of some of the less obvious developments of this disease; among these were the cases in which the only symptom is a peculiar barking or some other inhuman sound produced by irregular action of the laryngeal muscles; or a peculiar and violent spasm of the orbicularis, most frequent in old age, which makes the patient wink violently; another was that of a woman whose head turned slowly over to the right shoulder, and remained there until guided back with the finger placed against the chin, and returned immediately in the same manner when the finger was removed.

With regard to the *course* of chorea, Dr. Dickinson said that, unlike the specific fevers, it had no tendency to end within a definite time, and was not generally fatal. The cases which do end fatally were said to be almost always those in which the movements produce excoriations on the prominent parts of the body which they would not allow to heal, and perhaps also keep the patient from sleep. Under these circumstances, he would be found to fall into a condition which is characterized by dryness of tongue and rapid pulse, and differs only from that which results in typhoid fever in the skin remaining cool.

Dr. Dickinson professed himself unable to throw any new light on the *pathology* of this disease, other than that which might be yielded by one of the specimens he was about to show. He was inclined to attribute the cardiac complications of chorea to the hyperfibrinous condition of blood already alluded to, rather than to a genuine inflammation. They were unquestionably liable to occur, no matter from what exciting cause the disease arose, whether from fright, worms, or a loaded condition of the bowel, and whether or not it followed or was accompanied by distinct rheumatic affections.

As to *treatment*, his faith in most of the drugs of which trial had been made was very limited. Most patients improved more or less during the first fourteen days, if merely placed in bed and kept at rest; but if within this time recovery did not take place, medicinal treatment must be resorted to. First, it would be well to give a sharp purgative of calomel and jalap to insure the removal of sources of intestinal irritation, and then one of the metals which experience had shown to be of value. Of these, the most useful were the sulphate of zinc and iron in some form, the former applicable to florid and robust patients, and the latter to those who are anæmic. The administration of the salt of zinc should commence in doses of one grain every six hours, which should be increased by a grain daily up to from twenty to twenty-five grains. When thus administered, the emetic effect was avoided, and the majority of the cases to which it was applicable would recover. But if, on the contrary, the case should become chronic, a nervine tonic would be demanded; and of these the best were arsenic and strychnia; with these might be advantageously combined change of air and gymnastic movements; and in those cases which presented an hysteric element, the sudden application of cold, as, for example, by means of the shower-bath. Speaking of music, which has been said to be of value in the treatment of the Tarantula dance, Dr. Dickinson said that the connexion existing between the sense of hearing and the action of the voluntary muscles suggested an explanation of its possible value, and he regretted that it had not yet been fairly tested in this country.

Of the many other drugs which had been suggested for the treatment of chorea, Dr. Dickinson said there were none he could recommend. Sufficient trial had not been made of chloral in the hospital to enable him to pronounce upon it. Antimony certainly had a tendency to effect a marked diminution in choreic movements if given in large doses; but as chorea, when fatal, proves to be so through prostration, he considered that the use of so lowering a remedy must be unadvisable. Several of the fatal cases which had come within his cognizance had been treated with that metal. Belladonna he had seen given in daily quantities ranging up to seventy grains of the extract without any beneficial effect. He had no faith in either conia or bromide of potassium; and valerian was only of use where chorea is complicated with hysteria.

After the lecture, Dr. Dickinson exhibited a number of microscopic specimens taken from a child who had died of chorea. They showed that the pia mater, the corpus striatum, the pons Varolii, the velum, and the spinal cord—in fact, the entire cerebro-spinal nervous system—was loaded with blood-corpuscles; but the most interesting was a specimen which presented a small cavity in one of the anterior cornua of a portion of spinal cord taken from the lumbar region; the tissues at the circumference of the cavity were softened and broken, and the cavity itself was crowded with blood-corpuscles.

